# Enhancing Surgical Efficiency and Radiological Outcomes Through Advances in Patient-Specific Instrument Design

**DOI:** 10.3390/jcm14020307

**Published:** 2025-01-07

**Authors:** Yong-Gon Koh, Ji-Hoon Nam, Jong-Keun Kim, Dong-Suk Suh, Jai Hyun Chung, Kwan Kyu Park, Kyoung-Tak Kang

**Affiliations:** 1 Joint Reconstruction Center, Department of Orthopaedic Surgery, Yonsei Sarang Hospital, Seoul 06702, Republic of Korea; osygkoh@gmail.com (Y.-G.K.); osdssuh@gmail.com (D.-S.S.); jjhsky0843@gmail.com (J.H.C.); 2 Department of Mechanical Engineering, Yonsei University, Seoul 03722, Republic of Korea; 2018njh@gmail.com; 3 Skyve R&D LAB, Skyve Co., Ltd., Seoul 06698, Republic of Korea; 4 Department of Orthopaedic Surgery, Heung K Hospital, Gyeonggi-do, Siheung-s 14999, Republic of Korea; edmandoo@naver.com; 5 Department of Orthopedic Surgery, Severance Hospital, Yonsei University College of Medicine, Seoul 03722, Republic of Korea

**Keywords:** patient-specific instrument, total knee arthroplasty, knee, surgical time

## Abstract

**Background/Objectives**: Patient-specific instrumentation (PSI) in total knee arthroplasty (TKA) uses preoperative three-dimensional imaging to create cutting blocks tailored to patient anatomy. However, there is debate regarding the additional benefits of PSI in terms of improved alignment or functional outcomes compared to using conventional instruments. Although PSI design has undergone continuous development, the improvements have not been incorporated. Therefore, the aim of this study was to compare the surgical time and radiological outcomes between advanced-design PSI and conventional instruments. **Methods**: We conducted a retrospective review of 328 patients who underwent primary TKAs using PSI for osteoarthritis and compared them with 328 matched patients who underwent TKA performed with conventional instruments during the same period (March 2023 to August 2024). We compared the surgical time and component alignment between the advanced-design PSI group and the conventional instrument group. **Results**: The average surgical time was significantly shorter in the advanced-design PSI group (47.6 ± 12.4 min) compared to the conventional instrument group (59.2 ± 14.2 min, *p* < 0.05). The advanced PSI design group had a significantly lower occurrence of outliers in hip–knee–ankle alignment (7%) compared to the conventional instrument group (36.3%). This trend was also observed in femoral coronal alignment, tibial coronal alignment, and femoral sagittal alignment. **Conclusions**: The use of advanced-design PSI demonstrated significantly reduced surgical time and improved alignment compared to conventional instruments. This highlights that proper design is a key factor for PSI to achieve superior biomechanical effects. Our study shows that advanced-design PSI technology has the potential to replace conventional instruments in TKA, though further research is required to determine its clinical outcomes and economic benefits.

## 1. Introduction

Total knee arthroplasty (TKA) is a highly successful procedure that has been shown to restore knee function and improve clinical and functional outcomes in osteoarthritic knees. Despite advancements in surgical techniques and implant designs that have improved survival rates in TKA, early implant failure remains a concern [1,2]. Malignment of femoral and tibial components can lead to suboptimal outcomes, including pain, reduced range of motion, and early loosening [3,4].

In recent years, various technologies have been developed to enhance alignment in TKA, with computer-assisted surgical navigation demonstrating improved accuracy and precision in implant and neutral alignment [5]. Patient-specific instrumentation (PSI) has been designed to assist surgeons in achieving more precise prosthesis alignment, simplifying intraoperative decision-making, and reducing surgical time in TKA [6]. However, published reports indicate that, although some studies have demonstrated improved alignment [7,8], others have found no significant differences in accuracy compared to conventional instrumentation [9,10]. The first issue is determining whether MRI or CT is more appropriate when creating this PSI [11,12]. The main disadvantage of CT-based PSI is that it does not account for cartilage, requiring the creation of a cartilage-considering spike for the guide, which may lead to instability when contacting the bone [11]. Hence, despite the longer imaging time of MRI, its ability to produce highly accurate 3D models makes it a better option for PSI fabrication [11]. In addition, recent studies have demonstrated that modifying the design of PSI guides leads to significant improvements in surgical times and mechanical alignment restoration compared to conventional instruments [13,14].

Therefore, the aim of this study was to compare the surgical time and radiological outcomes between advanced-design PSI and conventional instruments. We hypothesize that advanced-design PSI shows improved surgical time and alignment compared to conventional instruments.

## 2. Materials and Methods

### 2.1. Study Design

Institutional review board approval was obtained. We retrospectively reviewed data from 328 consecutive patients who underwent TKA using an MRI-based advanced-design PSI system in a nonrandomized manner between March 2023 and August 2024. The inclusion criteria were a diagnosis of primary knee osteoarthritis and the capability to undergo MRI at our facility. The exclusion criteria were patients with previous osteotomy, infections, fractures, or defects in the distal femoral or proximal tibial regions requiring metal or allograft augmentation, or femoral or tibial stem extensions, as these could affect the radiographic interpretation of alignment achieved with each surgical technique [14]. We subsequently selected a matched cohort of 328 consecutive TKAs performed with conventional instruments as the control group. The groups were matched by age and BMI. The PSI group comprised 233 females and 95 males, while the conventional instrument group consisted of 268 males and 60 females.

### 2.2. Pre-Planning and PSI Design Methods

The advanced-design PSI group used the PNK total knee system (Skyve, Seoul, Republic of Korea), while the conventional instrument group used the Attune total knee system (DePuy Synthes, Warsaw, IN, USA). Preoperatively, patients underwent routine radiographic evaluation based on the treating surgeon’s standard clinical practice, along with MRI scans of the knee, hip, and ankle performed 2 weeks prior to surgery, following a PSI protocol. In the PSI group, an MRI-based PSI system Kneevigate 1.0 (Skyve) was utilized. The raw images were sent to Skyve for validation and then uploaded to the 3D surgical planning software OnKneeU 1.0 (Skyve). OnKneeU is a virtual knee surgery software system designed to effectively perform the entire process, from generating landmarks of the femur and tibia to inserting the artificial joint.

MRI scans were acquired using a 3.0-T MRI scanner (uMR 770; United-Imaging Healthcare, Shanghai, China). The MRI scans were obtained in a 1 mm slice thickness on the sagittal plane for the tibiofemoral knee joint and a 5 mm slice thickness on the axial plane for the hip and ankle joints. For the non-fat saturation condition, the MRI consisted of an axial T1-weighted fast spin–echo sequence. A high-resolution setting was used for the T2 gradient echo train imaging. The data were transferred using the digital imaging and communications in medicine format, and 3D reconstruction was carried out using Zift 1.0 (AIES, Daejeon, Republic of Korea). The femoral and tibial bone structures, along with the articular cartilage, were semi-automatically segmented. A 3D model was created based on the sectional MRI slices, using a masking process enhanced by the fill inner cell function.

**Figure 1:**
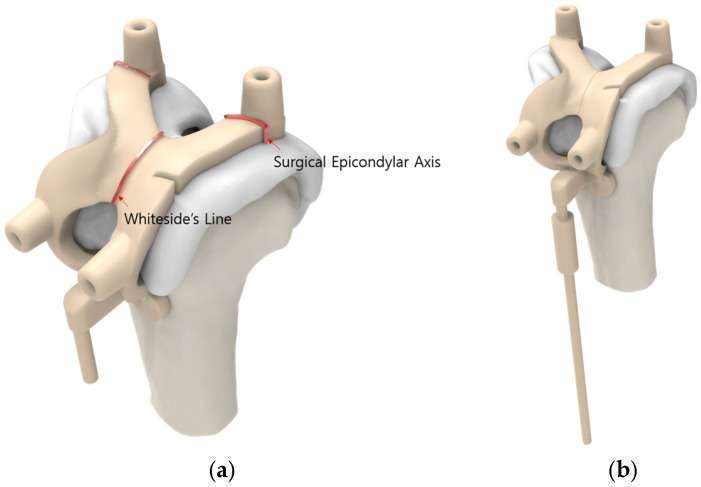

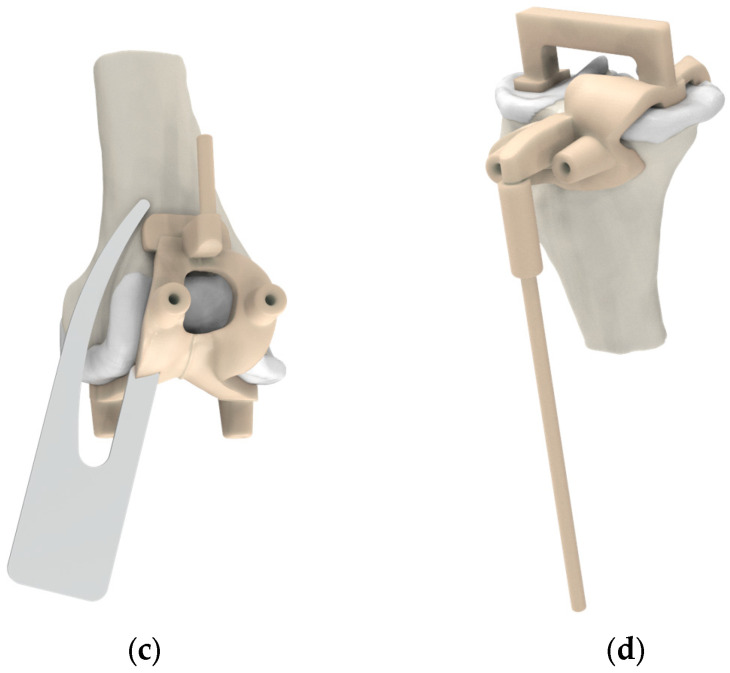
Advanced-design PSI components: (**a**) Rotation axis, (**b**) alignment rod, (**c**) notch check, and (**d**) tibial alignment rod.

**Figure 2:**
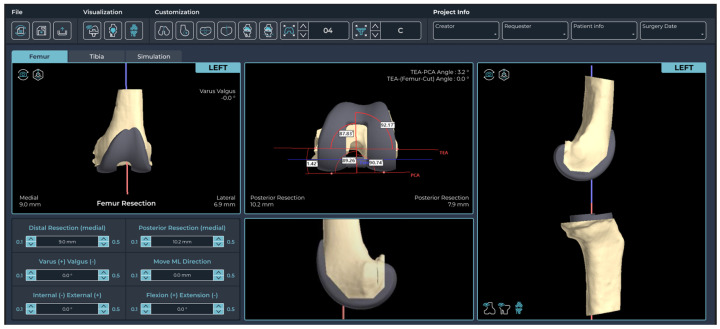
Planning interface of the Kneesign.

The advanced-design PSI group offers several advantages (Figure 1). First, the femoral guide is designed to contact the anterior flange, preventing both translation and rotation of the guide. Second, the PSI femoral guide includes a slot for direct verification of the notch and a mechanical alignment checker rod, offering the advantage of reduced surgical time. Additionally, the femoral guide is marked to allow verification of the surgical epicondylar axis and Whiteside’s line. The tibial guide design incorporates an additional contact point on the posterior proximal tibia and features a mechanical alignment checker rod. The 3D preoperative plan was developed based on the following surgeon preferences: the femoral component was aligned with the surgical epicondylar axis for rotational alignment, set at 90° to the mechanical axis in the coronal plane and positioned with 3° of flexion in the sagittal plane, along with a 9 mm distal medial resection. For the tibial component, the default rotational alignment was set to 0° relative to the anterior–posterior axis, with a coronal alignment of 90° to the mechanical axis and a sagittal alignment featuring a 5° posterior slope, accompanied by an 8 mm resection below the highest point of the lateral plateau [14,15,16]. For the conventional instrument group, the surgery was performed with intramedullary alignment for the femoral component. and extramedullary alignment for the tibial component. All the procedures were performed by three experienced knee replacement surgeons (Y-G Koh, D-S Suh, and J-H Jung) at a single institution. The surgeon checked and adjusted the sizing and positioning of the components at every step of the preliminary planning using Kneesign 1.0 (Skyve) (Figure 2).

### 2.3. Outcome Measurements

**Figure 3:**
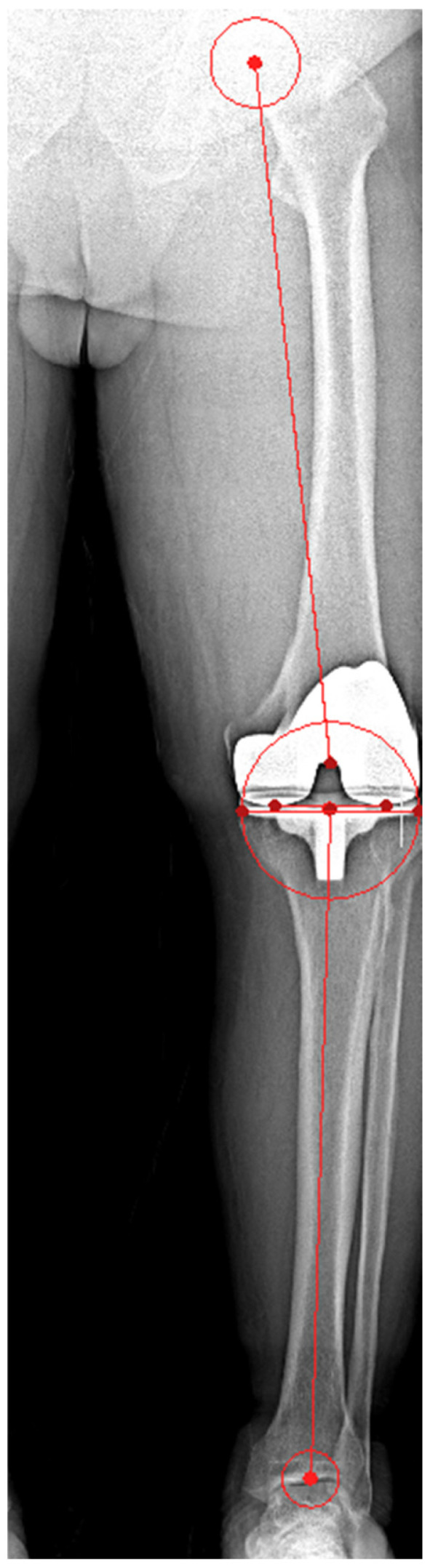
Illustration of coronal alignment parameters, including HKA, FCA, TCA.

**Figure 4:**
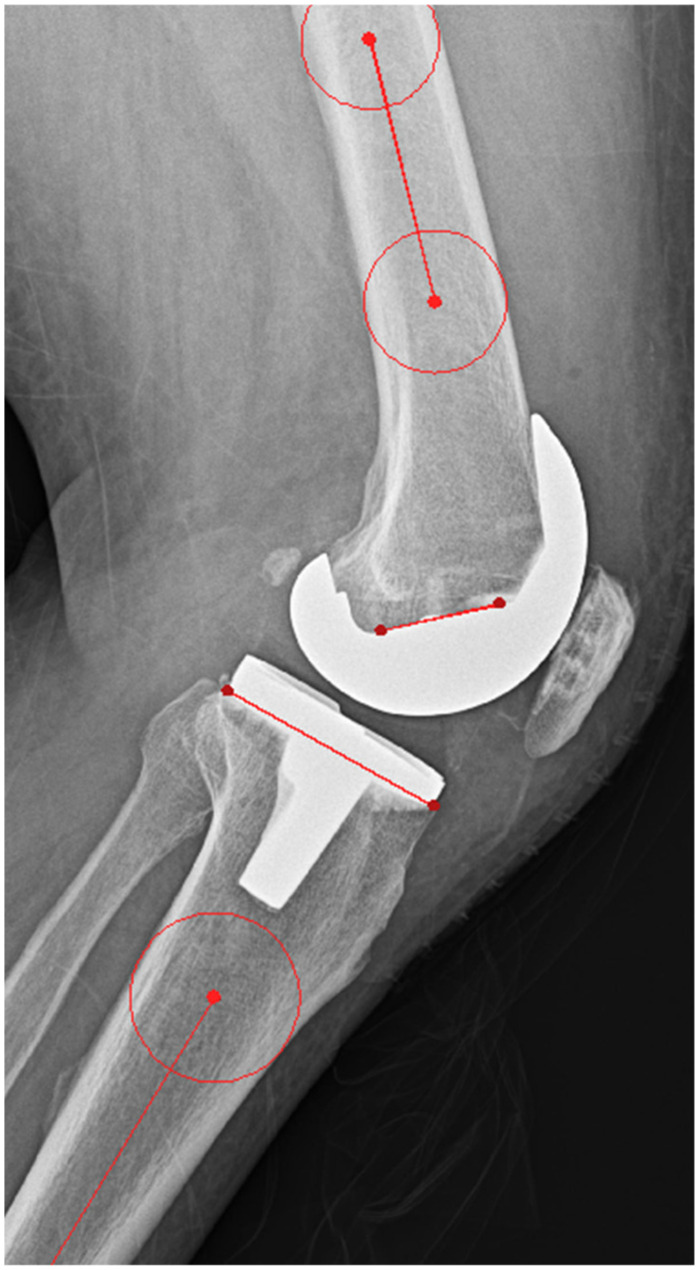
Illustration of sagittal alignment parameters, including TSA and FSA.

Full-length weight-bearing anteroposterior (AP) radiographs of the entire lower limb, including the femoral head, tibia, and talus, were obtained within 3 months following the index procedure. These were used to evaluate the postoperative mechanical axis in the coronal plane. The mechanical axis of the lower limb was measured on standing AP radiographs with the patella facing forward, using Mimics 20 (Materialise, Leuven, Belgium). The hip–knee–ankle (HKA) angle was measured as the angle between the femoral mechanical axis (FMA) and the tibial mechanical axis (TMA) (Figure 3). Since the HKA angle was measured from the medial side, an HKA angle of <180° indicates varus, while an angle > 180° indicates valgus. The femoral coronal angle (FCA) was measured as the angle between the FMA and the line tangent to the distal condyle of the femoral component, assessed medially. An FCA angle of <90° is defined as varus, while an angle of >90° is defined as valgus. The tibial coronal angle (TCA) was measured as the angle between the TMA and the line tangent to the tibial component, assessed medially (Figure 3). An TCA angle of <90° is defined as varus, while an angle of >90° is defined as valgus. In the sagittal plane, the tibial slope angle (TSA) and the flexion angle of the femoral component (FSA) were measured on lateral knee radiographs (Figure 4). Deviations exceeding 3° from the neutral mechanical axis were considered outliers, and the respective proportions were calculated. The evaluation was performed by the same medical team that conducted the surgical procedures.

### 2.4. Statistics

Continuous variables are presented as mean and standard deviation, while categorical variables are expressed as numbers and percentages. An independent *t*-test was used to compare the means between the two groups. For categorical variables, a chi-square test was performed. The significance level was set at 0.05. Statistical analyses were conducted using R (R Foundation for Statistical Computing, Vienna, Austria). A power analysis was performed using HKA outliers, resulting in a power of 100%. All measurements were performed twice at regular intervals by 2 experienced imaging specialists. The intra-observer reliability was 1, and the inter-observer reliability was 0.86.

## 3. Results

The mean age of the advanced-design PSI group was 70.5 ± 7.0 years, while that of the conventional instrument group was 69.7 ± 7.6 years. The mean body mass index (BMI) of the advanced-design PSI group was 26.8 ± 10.1, while that of the conventional instrument group was 26.8 ± 15.6 (Table 1). There were no statistically significant differences in demographic data between the two groups. The mean surgical time for the advanced-design PSI group was 47.6 ± 12.4 min, while it was 59.2 ± 14.2 min for the conventional instrument group. The advanced-design PSI group had a significantly shorter surgical time compared to the conventional instrument group (*p* < 0.05) (Table 2). Table 3 shows the radiological evaluation results of outliers along with statistical comparison of HKA, FCA, TCA, FSA, and TSA. There was a statistically significant difference in component alignment for HKA, FCA, TCA, and TSA between the two groups. However, there was no significant difference in FCA between the advanced-design PSI group and the conventional group (*p* = 0.453). The results of the outliers in component alignment did not match the component alignment measurements.

In the postoperative frontal alignment analysis, the proportion of patients with HKA malalignment ±3° was 7% in the advanced-design PSI group and 36% in the conventional instrument group (*p* < 0.05). For FCA, contrary to the measurement results, the outlier rate was 4.3% in the advanced-design PSI group and 11.9% in the conventional instrument group, with the rate in the advanced-design PSI group significantly lower than that in the conventional instrument group (*p* < 0.05). The opposite trend was observed in the TSA outliers. For TSA, although the measurement results showed a statistically significant difference, outlier analysis revealed no significant difference between the 2 groups (*p* = 0.117).

For TCA and FSA, the proportions of malalignment ± 3° were 7% and 32% in the advanced-design PSI group, respectively, compared to 12% and 57% in the conventional instrument group. For TCA and FSA, the advanced-design PSI group showed significantly lower rates compared to the conventional instrument group (*p* < 0.05).

## 4. Discussion

The most important finding of this study is that the advanced-design PSI group demonstrated a significantly shorter surgical time and significantly fewer outliers in radiological evaluations compared to the conventional instrument group. Therefore, our hypothesis was validated.

Innovations in TKA are constantly being developed to enhance surgical outcomes, ensure patient safety, improve efficiency, and optimize cost-effectiveness [15]. Technological innovations designed to improve limb alignment and component positioning include navigation surgery, robotic surgery, and PSI [16]. The major drawbacks of computer-assisted surgical navigation, including navigation and robotic systems, are difficulties in accurately registering intraoperative landmarks, longer setup and operative times, higher perioperative costs, risks of pin loosening and pin-site fractures, and a significant learning curve [17,18]. PSI is designed to enhance surgical precision and alignment without requiring intraoperative landmarks and theoretically offers the advantage of reducing surgical time, although 3D-printed PSI has the drawback of a preparation time of 2–3 weeks for its design and manufacturing [19].

However, as mentioned in the Introduction, there is ongoing debate regarding the functional outcomes of PSI. The authors believe that these reasons include the use of inconsistent imaging modalities, the learning curve, and the lack of consideration for advanced designs.

Most PSI manufacturers can produce PSI using either CT or MRI. While CT offers the advantage of shorter imaging times compared to MRI, it has a significant drawback of not accounting for cartilage [11]. According to Pfitzner et al. [20], MRI-based PSI is more accurate than CT-based PSI in terms of the coronal mechanical limb axis. A recent systematic review and meta-analysis revealed that alignment achieved with MRI-based PSI is not inferior to, and may even be superior to, that achieved with CT-based PSI. The study suggests that MRI should be the preferred imaging modality when performing TKA surgery with PSI to prevent malalignment. Another issue is the learning curve and the design of PSI, which are closely related. Theoretically, PSI surgery should not take longer than conventional instrument surgery because it omits the steps for intramedullary alignment of the femoral component and extramedullary alignment, with rotation and alignment of the components being directly determined. In a study by Hampton et al., the tourniquet time was recorded and found to be similar between the two groups (76 ± 5 min in the conventional instrumentation group and 74 ± 4 min in the PSI group) [21]. In contrast, Tammachote et al. showed that the PSI group had a surgical time 11 min shorter than that of the conventional group [22]. The reason for this is that surgeons new to PSI often do not fully trust the system and tend to double-check, resulting in an increase in surgical time. The authors believe this issue can be addressed through improvements in PSI design. In a previous study, we reported that improvements in the design of the PSI tibial guide resulted in a surgical time that was 18 min shorter than the conventional instrument group and 13 min shorter than the initial PSI design [13]. In a previous study, we demonstrated that incorporating an alignment checker rod into the tibial guide design can significantly reduce surgical time [13]. With the initial design of PSI, inexperienced surgeons, even after drilling the location for bone cutting using the PSI, would attach an extramedullary rod to the cutting block during surgery to verify the accuracy of the PSI guide. However, our advanced-design PSI includes an alignment checker rod, allowing real-time verification of the coronal alignment of the femoral and tibial components during surgery. Additionally, the femoral guide includes markers for verifying the rotational alignment of the femoral component, as well as a slot for checking the notch. These advancements in PSI design were able to reduce surgical time by an additional 16 min compared to our previous study [13].

The advantages of the advanced-design PSI were also confirmed through the outlier results of component alignment in radiographic evaluations. Our results in the outliers of HKA show noteworthy findings. In a recent study by Leeuwen et al., the proportion of malalignment ± 3° in HKA was 26%, while Huijbregts et al. reported 13%, both of which are higher than our proportion 7% [9,23]. The tendency for our component outliers to be fewer than those reported in previous studies was also observed in other aspects of coronal alignment. In our coronal alignment analysis, the outliers for the femoral component and tibial component were 4.3% and 7%, respectively. In the previous study by Pourgiezis et al. [24], the outliers for FCA and TCA were 6.7% and 22%, respectively, while in Abane et al.’s study [25], the outliers were 32% and 11%, respectively. These results highlight the importance of design in PSI. However, in TSA, there was no statistically significant difference between the advanced-design PSI and the conventional instrument group in terms of outliers. Previous studies have shown that the primary benefit of PSI guides in TKA is improved coronal alignment, generally considered to fall within 3° varus/valgus of the mechanical axis [26]. The three degree leeway is arbitrary and derived from acceptable radiographic measurement error. Logically, any deviation from the theoretical neutral limb alignment can reasonably be expected to shorten the longevity of TKA in proportion to the degree of malalignment [26,27].

As mentioned earlier, achieving a neutral mechanical axis is a key factor of survivorship in TKA [27]. Coronal alignment is particularly regarded as the gold standard for approximating the mechanical axis after TKA [20]. Nonetheless, the optimal sagittal alignment is still not well understood [28]. A PSI guide is theoretically expected to enhance the accuracy of limb alignment by guiding critical bone cuts to the ideal position for each patient [26]. Compared to conventional instruments, PSI guides are thought to better overcome errors caused by extramedullary deformities, large bone canals, patient obesity, and other anatomical variations [26].

Our results demonstrated a significant reduction in surgical time and improved accuracy in the mechanical axis as well as the coronal alignment of the femoral and tibial components. In TSA, although the results did not show a reduction in outliers compared to conventional instruments, further research could lead to modifications in the guide to improve sagittal alignment. In conclusion, our advanced-design PSI demonstrated superior outcomes compared to previous studies. This can be attributed, first, to the improved design of the guide, and, second, to the accuracy of the surgical plan and the precision of the pin placement. Preoperative planning is crucial for achieving optimal results, and careful evaluation and necessary adjustments to the provided PSI are essential [29]. Previous studies using the same PSI technology demonstrated that modifications during the planning phase were necessary in most cases [30]. Our PSI system (Kneesign) is based on a web-based platform, enabling easy, convenient, and quick surgical planning adjustments.

This study has three limitations. First, we used a nonrandomized design, with group allocation determined by patient preference. Retrospective studies inherently rely on pre-existing data, which can be subject to selection bias and incomplete information. Additionally, patient-preference allocation may lead to nonrandomized grouping, potentially influencing outcomes based on baseline differences between groups. Second, it does not include clinical assessment or long-term clinical outcomes. Third, the TKA products used in the PSI group differed from those used with conventional instruments. Fourth, we did not address the economic or logistical issues related to PSI.

## 5. Conclusions

Our study results demonstrate that using advanced-design PSI significantly reduced surgical time and improved alignment compared to conventional instruments. This highlights that design is a key factor for PSI to achieve superior biomechanical effects. Our study shows that advanced-design PSI technology has the potential to replace conventional instruments in TKA, though further research is required to determine its long-term clinical outcomes and economic benefits.

## Figures and Tables

**Table 1:** Age and BMI data.

Variables	Conventional Group	PSI Group	*p*-Value
Age (yrs)	69.7 ± 7.6	70.5 ± 7.0	0.07
BMI (kg/m^2^)	26.8 ± 15.6	26.8 ± 10.1	0.99

**Table 2:** Surgical time.

Variables	Conventional Group	PSI Group	*p*-Value
Surgical time (min)	59.2 ± 14.2	47.6 ± 12.4	<0.001

**Table 3:** Postoperative HKA and component alignment data with outliers.

Variables	Conventional Group	PSI Group	*p*-Value
HKA angle			
Value (°)	59.2 ± 14.2	47.6 ± 12.4	0.004
Outlier (n, %)	119 (36.3)	23 (7)	<0.001
FCA			
Value (°)	89.7 ± 1.9	89.8 ± 1.5	0.453
Outlier (n, %)	39 (11.9)	14 (4.3)	0.002
TCA			
Value (°)	89.1 ± 1.9	89.7 ± 1.6	<0.001
Outlier (n, %)	42 (12.8)	23 (7)	0.03
FSA			
Value (°)	85.0 ± 4.4	86.9 ± 3.0	<0.001
Outlier (n, %)	189 (57.6)	108 (32.9)	<0.001
TSA			
Value (°)	85.0 ± 4.4	86.9 ± 3.0	<0.001
Outlier (n, %)	95 (29)	123 (37.5)	0.12

## Data Availability

The data presented in this study are available on request from the corresponding author.
